# Editorialets

**Published:** 1907-01

**Authors:** 


### Editorialets.

HAPPY NEW YEAR, DOCTOR! I wish I could come around and shake every one of your fellows' hands, and wish you good luck.

DR. W. A. HARPER, eye, ear, nose and throat, Austin, Texas, has removed to his new offices, No. 900 Congress avenue.

F. E. DANIEL, M. D., "Red Back," Austin, Texas. Dear Doctor: Kindly send me statement of my account. Although out of active practice, and a kind of M. D. *non est,* still I can't do business without the "Red Back." Fraternally yours,

GEO. F. PERRY, Hamilton, Texas.

DR. GEO. R. TABOR. In retiring from the office of State Health Office Dr. Tabor carries with him the confidence and esteem of not only the entire medical profession of Texas and the Southwest, but of the immense business interest of the country. His administration, extending over about five years, was clean, ethical, on an advanced plane. It was efficient, vigorous and satisfactory. Never a word of complaint has been uttered against it.

Dr. Tabor will resume the practice of medicine and will remain in Austin. "Well done thou good and faithful (and courageous) servant," is the verdict of the people, lay and professional.

---

THE name of the *Journal of the Association of Military Surgeons* is changed with the issue for January, 1907, to *The Military Surgeon,* retaining the old name as a subsidiary title. We have noted the highly successful career of this journal during the past six years. It is the pioneer military medical journal in the English language, occupying a field hitherto unexplored. Its success in creating a reliable body of military medical literature was not unexpected, although it has certainly been remarkable. We congratulate our esteemed colleague, Dr. James Evelyn Pitcher, the brilliant and gifted editor.

---

WANTED. Location in Texas or New Mexico by an eye and ear specialist and expert refractionist. Will sell or exchange fine home and sanitarium for farm or town property in the South. Address, J. L. SHORT, M. D., Versailles, Mo.

---

DEATH OF DR. MACDONALD. Dr. A. E. Macdonald, late superintendent of the Manhattan (N. Y. State) Insane Asylum, and ex-President of the American Medico-Psychological Association, died at his home in New York of consumption December 7, ult. Dr. Macdonald was one of the ablest and most distinguished alienists in America. His death is greatly deplored. It seems he contracted consumption on his trip to Texas and Mexico in the spring of 1905, and it ran a very rapid course; death came suddenly and much earlier than was expected.

---

PHYSICIANS who are interested in the study and legitimate practice of the physical (drugless) therapeutic methods, notably electrotherapy, phototherapy, mechano-therapy, hydrotherapy, suggestion and dietetics, are invited to join the American Physio-Therapeutic Association. Address the secretary, Dr. Otto Juettner, No. 8 W. Ninth St., Cincinnati, O. The officers for the ensuing year are: President, Dr. H. H. Roberts, Lexington, Ky.; Secretary,

Dr. Otto Juettner, Cincinnati, O.; Treasurer, Dr. Geo. H. Grant, Richmond, Ind.; Executive Council, Drs. W. F. Klein, Lebanon, Pa.; Jas. Hanks, Brashear, Mo.; J. W. Unger, West Point, Miss.; Chas. S. Northen, Talladega, Ala.; R. W. Gibbes, Columbia, S. C.; S. J. Crumbine, Topeka, Kan.; A. L. Blesh, Guthrie, Okla.

---

*The American Journal of Dermatology and Genito-Urinary Diseases,* St. Louis, has been enlarged and in many ways improved. It numbers amongst its contributors some of the most celebrated dermatologists, syphilologists and genito-urinary surgeons who write for journals, and every general practitioner will find in its pages much to help him in this line of ailments. Success to our worthy confrere.

---

"The Antiphlogistine Girl — An Angel of Mercy." We have received with the compliments of The Denver Chemical Company, New York, a framed photograph of a pretty trained nurse in the act of opening a can of Antiphlogistine to apply to some fellow with a terrible pain: sure relief. Thanks, very much. It is quite a valuable addition to my gallery of art.

---

In children suspected of having a foreign body in the larynx it will be generally found necessary to anesthetize the patient before a satisfactory laryngoscopic examination can be made, although in the presence of severe dyspnea no time should be lost in performing tracheotomy.—*International Journal of Surgery.*

---

That's It. The New York Pharmaceutical Company, in renewing its advertising contract for 1907 (paying a year in advance every year), writes: "We are limiting our appropriation for journal advertising, but we desire to renew with the Texas Medical Journal. The retaining of your journal on our list is an expression of our appreciation of the value of your publication as an advertising medium. Wishing you and the Journal a prosperous new year, we are, yours very truly."

---

Dr. T. H. Harrell, formerly of Hutto, Texas, more recently of Pueblo, Mexico, has been appointed chief surgeon of the Oaxaca Smelting, Mining and Railroad Company, and is now at the San Juan mines, Ocotlan, Oaxaca, Mexico. He will superintend building a large modern hospital for the company at that place and will have headquarters at Oaxaca City.

Dr. P. M. Payne has removed from Quinlan, Texas, to Rascon, San Luis Potosi, Mexico.

---

Dr. Worsham Reappointed. (January 18.) It is now known that Dr. B. M. Worsham, the popular and efficient Superintendent of the State Lunatic Asylum at Austin, and the creator of the Pasteur Institute, will be retained in that position. This meets the unqualified endorsement of the medical profession. Changes in the other State medical institutions have not yet been announced.

---

The American League for the Prevention and Cure of Tuberculosis has been making an exhibit in Mexico of pathological specimens, maps, etc., and during the week of January 5th to 10th exhibited in San Antonio, Texas. A large crowd witnessed the exhibition. Dr. Carter of Galveston, Drs. Bleim, Paschal, Lankford and Moss of San Antonio, made addresses and discussed the question generally, which discussion was participated in by others of the local profession. Our colleague, Dr. M. M. Smith of Austin, also, by invitation delivered a lecture on "Home and Sanitarium Treatment of Consumption," which was much applauded and favorably commented on by the San Antonio papers. The exhibit returned to Washington, its headquarters.

---

For Sale. A small, clean, up-to-date stock of drugs; a good business established; the store for sale or rent. Address, Frank Ashley Drug Co., Rockett, Texas. H. W. Aldridge.

---

Dr. H. B. Granbury of Austin has been elected President of the Travis County Medical Society, and Dr. G. L. Robertson of Leander, President of the Austin District (Seventh Councillor's) Medical Society.

---

Eczema of the breast should always be viewed with suspicion, for it may be a symptom of Paget's disease and precursory to cancer. In these cases the growth may for a long time appear as a superficial ulcer, and thus lead to errors in diagnosis.—*International Journal of Surgery.*

---

In chronic mastitis the existence of pain and swelling of the glands in the axilla often awakens a suspicion of cancer. If the affected area is adherent to the skin and muscles, with puckering, the diagnosis may be impossible before a microscopic examination has been made.—*International Journal of Surgery.*

